# Prevalence of Temporomandibular Disorder Symptoms among Orthognathic Patients in Southern Germany: Retrospective Study

**DOI:** 10.1155/2018/4706487

**Published:** 2018-10-18

**Authors:** Amjad M. AlWarawreh, Zaid H. AlTamimi, Hazem M. Khraisat, Winfried Kretschmer

**Affiliations:** ^1^Royal Medical Services, Amman, Jordan; ^2^Royal Medical Services, Zarqa, Jordan; ^3^Maxillofacial Department, Paracelsus-Krankenhaus Ruit, Ostfildern, Germany

## Abstract

This study investigated the prevalence of temporomandibular disorder (TMD) among patients before and after orthognathic surgery and assessed the effect of orthognathic surgery on each of the TMD symptoms (clicking, pain, crepitus, and MRI findings). A sample of 100 consecutive patients undergoing bimaxillary surgery for correction of craniofacial deformities (31 male and 69 female), with ages ranging between 17 and 58 years (mean age: 27.7 ± 9.3 years), were interviewed and examined regarding signs and symptoms of TMD. Clinical examination and X-ray and magnetic resonance imaging of the temporomandibular junction were performed at the time of surgery and 1 year thereafter. The prevalence of TMD preoperatively and postoperatively was 35% and 27%, respectively. A high frequency of relief was found in the patients with TMD symptoms (74.3%; 19 (70.3%) of patients had reduced clicking, 7 (87.5%) patients had reduced pain, 4 (100%) patients had reduced crepitus, and 4 (57.1%) patients showed changes in MRI findings), 12 patients who were asymptomatic before surgery developed clicking in TMJ after surgery, 3 developed pain, and 3 developed crepitus. TMD problems can occur in a variety of patients, including those who have facial deformities, and require orthognathic surgery. However, orthognathic surgery may not predictably treat or reduce the symptoms of TMD.

## 1. Introduction

Temporomandibular disorder (TMD) is considered to be a common problem in the population, affecting individuals from adolescence to adulthood. Common symptoms of TMD are pain, headache, crepitus, and clicking of the temporomandibular joint (TMJ), limitation of mouth opening, and masticatory difficulty, among others.

Orthognathic surgery has become a widely used procedure due to the increased demand for this procedure for aesthetic reasons; however, the effect of the procedure on the temporomandibular joint (TMJ) and on the TMD problems remains controversial [1–3]. Some studies have shown relief or stabilizing effects on the signs and symptoms of TMD [4]. On the other hand, other studies have reported the risk of developing TMD symptoms in the patients that were asymptomatic preoperatively [5]. Thus, further investigation into the factors that affect the TMJ in the context of orthognathic surgery is required.

The objective of this retrospective study was to evaluate TMD symptoms before and after orthognathic surgery in the patients who underwent this procedure and to evaluate the effect of the surgery on symptomatic as well asymptomatic patients.

## 2. Materials and Methods

This retrospective study was performed on 100 consecutive orthognathic patients, who were referred to the Department of Maxillofacial and Reconstructive Surgery at the Paracelsus-Krankenhaus Ruit, Ostfildern, Germany, for orthognathic surgery between October 2011 and October 2014. All patients underwent the same surgical procedure for bimaxillary jaw correction (LeFort I in the maxilla and bilateral sagittal split osteotomy (BSSO) in the mandible) by four maxillofacial surgeons who were well qualified and supervised by the head of department (W, K) who checked every operation regarding reproducibility and following the same protocol to reduce bias of multiple operators. Only rigid osteosynthesis without intermaxillary fixation was applied. The maxillary osteotomies were stabilized by 4 microplates with 1.5 mm or 2.0 mm diameter screws. Fixation in the mandible was performed with 2 microplates and 2 mm diameter monocortical screws (class II) or 1 microplate and 1 bicortical screw (class III).

All patients underwent a full-clinical examination of TMJ before operation and 1 month after the operation and 1 year thereafter by one examiner (W. K). Full medical and dental histories were taken by trained maxillofacial specialists that followed the same examination protocol; additionally, a full investigation, including OPG and lateral cephalometric X-rays and computed tomography (CT) scans, was performed for every patient. In addition, magnetic resonance imaging (MRI) was performed presurgically for every patient with a history of TMD-related problems. Postsurgically, MRI was performed for every patient at one month and one year as standard medical care was followed in the hospital. All MRI were taken with a coil using 3.0 tesla MRI machine. All MRI reports were examined by the radiologist from the Radiology department in the same hospital.

The exclusion criteria were a history of craniofacial syndromes, systemic arthritis, and muscle diseases, and a dentition of fewer than 24 teeth, any unstable or relapsed cases which need redo were also excluded. The study was approved by the Ethics Committee of the Royal Medical Services. In addition, verbal consent was taken from each patient by the operator.

### 2.1. Statistical Analysis

Data were entered and coded using the Statistical Package for the Social Sciences software (SPSS version 17.0, Chicago, IL, USA) program. Multiresponse frequency and means (shown as mean ± s.d.) were determined, and frequencies are shown as simple bar graphs.

## 3. Results

Among the 100 patients included in our study, 31 were male and 69 were female, and the age range was 17−58 years (mean age: 27.7 ± 9.3 years); 47% of patients had skeletal class II malocclusion, while 53% had skeletal class III malocclusion.

**Figure 1:**
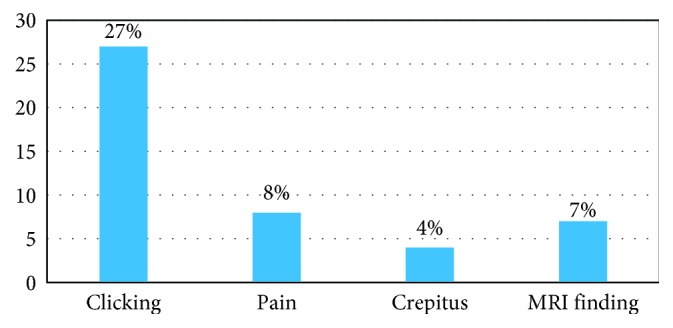
Distribution of preoperative temporomandibular disorder symptoms.

**Figure 2:**
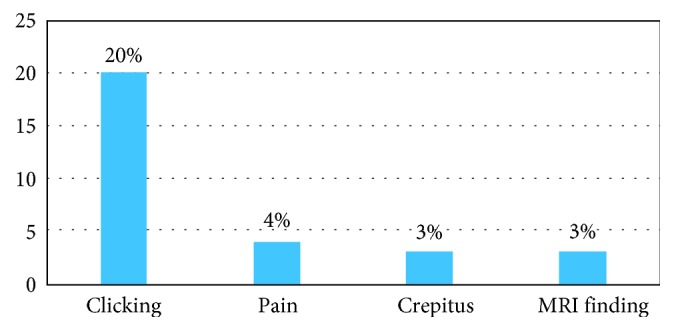
Distribution of postoperative temporomandibular disorder symptoms.

Before surgery, 35% of the patients (*n*=35) had 1 or more TMD problems: 27 patients (27%) had clicking, 8 (8%) had pain, 4 (4%) had crepitus, and 7 (7%) patients had indications of TMD on MRI (Figure 1). After surgery, 27% (27 patients) experienced 1 or more TMD problems, with 20 (20%) patients experiencing clicking, 4 (4%) experiencing pain, 3 (3%) showing crepitus, and 3 (3%) showing MRI changes indicative of TMD on MRI (Figure 2).

**Figure 3:**
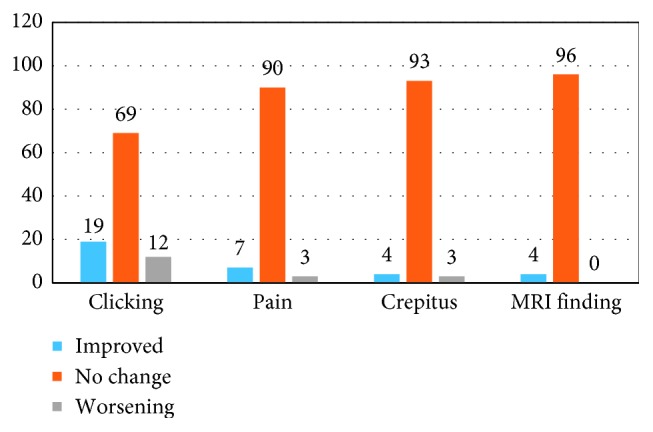
Effect of orthognathic surgery on the temporomandibular joint.

After surgery, 19 (19%), 7 (7%), 4 (4%), and 4 (4%) patients had improvement or relief of clicking, pain, crepitus, and MRI findings, respectively. On the other hand, among patients who had no symptoms prior to orthognathic surgery, but developed TMD problems thereafter, 12 (12%) developed clicking, 3 (3%) complained of pain, and 3 (3%) developed crepitus, although no MRI findings were found among the presurgically nonsymptomatic patients (Figure 3).

According to skeletal discrepancy, it was found that skeletal class II malocclusion with TMD symptoms before surgery was in 16 patients and 15 patients postsurgically. On the other hand, skeletal class III malocclusion with TMD symptoms before surgery was in 19 patients and 12 patients postsurgically (Table 1).

MRI shows disappearance of its finding in skeletal class II associated with disc displacement without reduction, but nothing else.

## 4. Discussion

We investigated the effect of a bimaxillary orthognathic surgery procedure on TMD symptoms. In our study, 35 (35%) of 100 patients presented with 1 or more symptoms of TMD preoperatively: 27% presented with clicking, 8% with pain, 4% with crepitus, and 7% showed MRI findings at the TMJ. A total of 65% were free of any symptoms, which was less than the proportion observed in previous studies by Dujoncquoy et al. [6], Karabouta and Martis [1], and White and Dolwick [7], i.e., 56.1%, 40.8%, and 49.3%, respectively. However, the proportion of the symptomatic patients was larger than that reported in a previous study by De Clercq et al. [8], i.e., 26.5% of patients were symptomatic for TMD before surgery.

In our study, 74.3% of patients with 1 or more TMD symptoms showed significant improvement after bimaxillary orthognathic surgery (19 (70.3%) patients improved in terms of clicking, 7 (87.5%) in terms of pain, 4 (100%) in terms of crepitus, and 4 (57.1%) in terms of MRI findings), which was similar to previous studies [1, 3, 6, 9]. Similarly, Dujoncquoy et al. [6] also reported that a high percentage (80%) of the symptomatic patients showed improvement after surgery (73.7% clicking, 43.8% pain, and 72.7% crepitus). Moreover, 7 patients showed relief from TMD pain after orthognathic surgery (87.5%) which was markedly higher that that reported previously. Dujoncquoy et al. reported an improvement in 43.8% of patients [6], while Wolford et al. reported that 84% of patients experienced TMD pain postsurgically compared to 36% presurgically [3]. Additionally, 4 patients (100%) experienced relief of TMJ crepitus after the surgery, which was higher than that reported in previous studies. This may have been due to the low number of patients included; yet, the proportion was high and agreed with the findings of Dujoncquoy et al. (72.7%). These results showed the benefit of bimaxillary orthognathic surgery, in agreement with previous studies [1, 9].

On the other hand, some asymptomatic patients developed symptoms after surgery, as follows: clicking developed in 12 of 73 asymptomatic patients (16.4%) and pain developed in 3 of 89 asymptomatic patients (3.3%), while crepitus developed in 3 of 96 patients (3.1%); these results agree with the literature [10]. Nevertheless, these results cannot be considered to be related only to the orthognathic surgery, as Panula et al. previously reported that 15% (*n*=3) patients from the control group developed such symptoms [11]. Thus, surgical management of TMD can occur independent of, or in conjunction with, orthognathic surgery [3]. Until an equilibrium between TMJ soft tissue and muscles is achieved, the patients undergoing orthognathic surgery should be informed of the possibility of the onset of TMD symptoms [12]. We used rigid fixation with plates for all patients without intermaxillary fixation; Buckley et al. showed no significant differences between using rigid fixation or nonrigid wire osteosynthesis in BSSO [13]. TMD symptoms are not affected by the type of fixation [14]. Previous studies have shown that the patients are generally highly satisfied after orthognathic surgery; this can probably be attributed to the major aesthetic changes, which prompt patients to pay less attention to TMD symptoms [15]. According to Onizawa et al., these changes are not due to correction of malocclusion, but rather due to the effects of the surgery on masticatory muscles [16]. Pahkala and Heino have shown that the patients with a primarily myogenous origin of TMD are more likely to derive benefit from this intervention than the patients in whom the condition has an isolated arthrogenous origin [17].

Regarding type of skeletal malocclusions, it was found that skeletal class II malocclusion (improved 11 patients and worsened 8 patients) showed more deterioration and developing TMD than skeletal class III malocclusion (improved 15 patients and worsened 7 patients) (Table 1). This is agreed with previous studies [11, 18].

Only skeletal class II associated with disc displacement without reduction shows improvement and disappearance of MRI finding nothing else, which needs further investigation.

Limitation of this study was incomplete MRI presurgically for all patients even without TMD symptoms, but due to medical care regulations and standards in the time of surgery, it was not done.

## 5. Conclusion

TMD problems can occur in various patients, including those who have facial deformities and require orthognathic surgery. Orthognathic surgery for treatment or reduction of the symptoms does not necessarily yield predictable results. Further investigations of skeletal malocclusion and the type of surgery used, as well as the degree of correction and its effect on the orofacial muscular system, require further clarification in future.

## Figures and Tables

**Table 1:** Distribution of patients regarding skeletal classification (class II and class III).

	Total	Presurgical patients with symptoms	Postsurgical patients with symptoms	Improved (improved/total patient ∗ 100%) (improved/total patients with TMD symptoms before surgery ∗ 100%)	Worsened (worsened/total patient ∗ 100%) (worsened/total patients without TMD symptoms before surgery ∗ 100%)
Class II	47	16	15	11 (23.4%) (68.8%)	8 (17.0%) (25.8%)
Class III	53	19	12	15 (28.3%) (78.9%)	7 (13.2%) (20.6%)
Total	100	35	27	26 (26.0%) (74.3%)	15 (15.0%) (23.1%)

## Data Availability

The data were collected from Maxillofacial Department, Paracelsus-Krankenhaus Ruit, Ostfildern, Germany, and they are available as Data Excel and SPSS Version.
